# Evolution of an intron-poor cluster of the CIPK gene family and expression in response to drought stress in soybean

**DOI:** 10.1038/srep28225

**Published:** 2016-06-17

**Authors:** Kaikai Zhu, Fei Chen, Jinyi Liu, Xinlu Chen, Tarek Hewezi, Zong-Ming (Max) Cheng

**Affiliations:** 1 Department of Plant Sciences, University of Tennessee, Knoxville, Tennessee 37996, USA

## Abstract

Calcium ion is an intracellular messenger that plays a central role in signal transduction pathways. Calcineurin B-like proteins (CBLs) and CBL-interacting protein kinases (CIPKs) signal network have shown different functions in the Ca^2+^ signaling process. In this work, we identified the entire soybean (*Glycine max*) CIPK gene family, which comprised 52 genes and divided into four subgroups (I to IV) based on phylogeny. The gene structural analysis separated these 52 genes into an intron-rich clade and an intron-poor clade. Chromosomal location analysis resulted in the identification of 22 duplicated blocks and six tandem duplication events. Phylogenetic classification of 193 CIPK proteins from representative plant species suggested that the intron-poor clade of CIPKs originated in seed plants. Analysis of global gene expression patterns of soybean CIPK family revealed that most intron-poor soybean CIPK genes are drought-inducible; a finding that was further confirmed using qRT-PCR. Our study provides a foundation for further functional analysis to reveal the roles that CIPKs and more specifically the intron-poor clade play in drought tolerance in soybean.

Soybean (*Glycine max*) is one of the most important legume crops for edible oil and protein source for human consumption and animal feed^1^. However, environmental factors such as drought, salt, heat and heavy metals may severely affect soybean growth and crop production^2^. Among those environmental stresses drought can severely inhibit all stages of soybean growth and productivity.

Global climate changes, driven by greenhouse gas emissions, can induce severe drought^3^,^4^. To cope with drought, higher plants have developed numerous mechanisms in responding to drought stress. Several genome-wide gene expression profiling pointed to a role of various protein kinase gene families in providing an efficient, fast-acting, and reversible response to drought stress^5^,^6^,^7^,^8^,^9^.

Calcium is an important signaling messenger in the course of plant response to environmental stresses and plant growth and development^10^. Four major Ca^2+^ sensors have been identified in *Arabidopsis*, including calcium-dependent protein kinase (CDPK), calcineurin B-like protein (CBL), calmodulin (CAM), and calmodulin-like protein (CML)^11^. With the exception of CDPKs, which contain a kinase domain, the other three Ca^2+^ sensors had no enzymatic domain, implying a role of their interactors in transmitting cellular signals to their downstream targets. CBLs modulate the activity of CBL-interacting protein kinases (CIPKs), also known as sucrose non-fermenting 1 related kinase 3 (SnRK3)^12^. CIPK family members frequently have a serine/threonine protein kinase domain in the N-terminus and a self-inhibitory NAF domain in C-terminus^13^. Activation of CIPKs is frequently mediated through the interaction of NAF domain with CBLs^14^.

The CBL and CIPK proteins form a calcium decoding signaling network and play an important role in plant responses to abiotic stresses. Among the 26 *CIPK* genes identified in *Arabidopsis*, several genes have been characterized^15^. For example, *AtCIPK24* (known as *Arabidopsis SOS2*) and *AtCBL4* (known as *Arabidopsis SOS3*) contribute to salt stress by activating the Na^+^/H^+^ antiporter, *AtSOS1* in order to maintain Na^+^ at low level^16^. AtCBL1 and AtCBL9 were reported to form a complex with AtCIPK23 to regulate potassium homeostasis under low potassium stress^17^. A role of AtCIPK7 in cold tolerance has been reported^18^. Certain CIPKs in other plant species have also been characterized. For example, overexpression of *OsCIPK23* improved rice drought tolerance by enhancing the expression level of drought-related genes^19^. A cotton *CIPK* gene, *GhCIPK6*, was induced by drought, and was found to enhance plant tolerance to drought when was overexpressed in *Arabidopsis*^20^. These findings suggest that CIPK family plays important roles bringing about plant tolerance to environmental stresses.

The CIPK gene family has been analyzed in *Arabidopsis*^21^,^22^, poplar (*Populus trichocarpa*)^22^, maize (*Zea mays*)^23^, rice (*Oryza sativa*)^24^, and canola (*Brassica napus*)^25^. These studies reported that the CIPK gene family could be divided into an intron-rich clade and an intron-poor clade, in which a subgroup in *Arabidopsis*, maize, rice and canola *CIPK* genes were induced by drought^23^,^24^,^25^,^26^. Although soybean (*Glycine max* var. Williams 82) has been sequenced^1^, no specific study has been reported on characterization of the CIPK gene family at the genome level to obtain a general perspective into their potential biological functions, especially in response to drought stress. In this study, we identified 52 soybean CIPK family members and found that intron-poor *CIPK* genes might be originated in seed plants. We also analyzed the gene expression patterns of the *CIPK* genes using publicly available microarray data and found that several *CIPK* genes are drought-inducible, from which 18 genes were confirmed using quantitative real-time polymerase chain reaction (qRT-PCR). Taken together, our data implicate soybean CIPK gene family in drought tolerance and point into several candidate genes for further functional characterization towards improving drought tolerance in soybean.

## Materials and Methods

### Genome-wide identification of CIPK gene family in soybean

To identify soybean CIPK proteins, all protein sequences were downloaded from the soybean genome (Wm82.a2.v1) from Phytozome V10 (http://phytozome.jgi.doe.gov/pz/portal.html#!info?alias=Org_Gmax). Hidden Markov Models were used to search for putative soybean CIPK proteins. A HMM profile of the NAF domain (PF03822), the signature domain of CIPKs, was first downloaded from Pfam (http://pfam.xfam.org/)^27^ and used to search for soybean CIPKs by HMMER 3.0^28^. Each CIPK candidate sequence was examined for the presence of the NAF domain and protein kinase domain to be considered as a member of soybean CIPK family. The putative CIPK family members were further examined using Pfam and SMART domain detection softwares (http://smart.embl-heidelberg.de/smart/set_mode.cgi?GENOMIC=1)^29^. Molecular weight (MW) and isoelectric point (pI) of each protein sequence were calculated using ExPASy (http://web.expasy.org/compute_pi/)^30^.

### Multiple alignment and phylogenetic tree construction

The protein sequences of all the 52 GmCIPK family members were aligned with ClustalX and constructed using Neighbor-Joining (NJ) method by MEGA6.06^31^. The bootstrap values for phylogenetic tree were based on 1000 replicates. Protein sequences from soybean, *Arabidopsis*, grape, rice, amborella, gymnosperm plants (pine, gnetum, ephedra, welwitschia and ginkgo), spikemoss, fern, moss and green algae were aligned with ClustalX. The phylogenetic tree was constructed with MEGA6.06 using the NJ method, and bootstrap analysis using 1000 replicates with the pairwise deletion and Poisson model.

### Exon-Intron structure analysis and identification of conserved motifs

Gene structure analysis of *GmCIPK* subgroup was performed using Gene Structure Display Server (GSDS 2.0, http://gsds.cbi.pku.edu.cn/)^32^ by aligning the cDNAs with the corresponding genomic DNA sequences. Motifs analysis was performed with the MEME program (http://meme-suite.org/tools/meme)^33^. The parameters were as follows: number of repetitions, any; maximum numbers of motifs, 30; and the optimum motif widths, between 6 and 200 residues.

### Chromosomal location and gene duplication

The chromosomal location image of soybean *CIPK* genes was generated by Mapchart 2.30 (www.wageningenur.nl/en/show/Mapchart-2.30.htm). The chromosomal position information of soybean *CIPK* genes was collected from the phytozome database (http://phytozome.jgi.doe.gov/pz/portal.html). Duplication patterns of *GmCIPK* genes were assigned based on their locations. The tandem duplicated genes were defined as an array of two or more genes located on the same chromosome and separated by five or fewer genes in a 100-kb region^34^. Genes located on duplicated chromosomal blocks were considered as segmental duplication. The information for segmental duplication was obtained from the SoyBase browser (http://soybase.org/gb2/gbrowse/gmax2.0/)^35^.

### Microarray analysis of soybean CIPK genes expression

Gene expression pattern of soybean CIPK gene family under drought stress, were downloaded from the Gene Expression Omnibus (GEO) database (http://www.ncbi.nlm.nih.gov/geo/) at the National Center for Biotechnology Information (NCBI). The two microarray data sets represented expressions of 48 *CIPK* genes in soybean leaves at a vegetative stage (GSE29663) and a reproductive stage (GSE40604) under drought. The heatmap of soybean CIPKs was generated using Multi experiment viewer 4.8 (MeV 4.8) software (http://www.tm4.org/mev.html)^36^.

### Plant materials, growth conditions and drought treatment

Soybean (var. Williams 82) was used in this study. Soybean seedlings were grown in a growth chamber at 25 °C with a photoperiod of 12 h/12 h, and a light intensity of 180 μmol m^−2^ s^−1^. The seedlings were watered every two days before drought treatment^20^. At 15 days post germination, drought treatment was initiated and this was set as the 0 day of drought treatment. Leaves, stems and roots samples were collected at 0, 4, 8 and 12 days, respectively, after as the initiation of drought stress (Figure S2). Control seedlings were continuously watered every two days. The collected samples were frozen in liquid nitrogen immediately and stored at −80 °C for further analysis. Three biological replicates, each contained three plants, were used for each treatment or control.

### RNA extraction and gene expression assay by qRT-PCR

Total RNA was isolated from leaves, stems and roots using the PureLink Plant RNA Reagent (Invitrogen, Carlsbad, CA) according to the manufacturer’s instructions. Then RNA was treated with DNase I (RNase-free DNase set, Qiagen, Hilden, Germany) to eliminate trace of DNA, and RNA quality and concentration were measured using NanoDrop 1000 (Thermo Scientific, Wilmington, DE). First strand cDNA was synthesized from 2 μg total RNA using cDNA reverse transcription kit (Applied Biosystems, Foster city, CA) with RNase Inhibitor (RNase out, Invitrogen Life Technologies, Carlsbad, CA) according to the manufacturer’s instructions. Specific primers for the 18 soybean *CIPK* genes (Table S1) were designed using Primer 3 software (http://bioinfo.ut.ee/primer3/). *Ribosomal protein s20e* gene (*RSP s20e*, *Glyma.03G142300*) was used as a reference gene^37^. The real-time qRT-PCR was conducted using a Power SYBR Green PCR Master Mix Kit (Applied Biosystems, Foster city, CA) on an ABI 7900HT Fast Real-Time PCR System (Applied Biosystems, Foster city, CA). The PCR reactions were performed according to the manufacturer’s protocol. The PCR conditions were as follows: 95 °C 10 min, 40 cycles of 15 sec at 95 °C and 1 min at 60 °C, at the end, the melting curve analysis was executed for verifying the specificity of the primer with the following stage: 95 °C for 15 sec, 60 °C for 1 min, 95 °C for 15 sec. Three biological replicates were used per treatment or control. Quantification of gene expression changes in stressed plants relative to control were performed using the 2^−ΔΔCT^ method^38^.

### Statistical analysis

Values are means ± SE of three different experiments with three replicated measurements. Statistical analysis was performed using Student’s *t* test (*P* < 0.05).

## Results

### Soybean genome encodes 52 *CIPK* genes

We identified 52 CIPK gene family members (GmCIPK1 to GmCIPK52) that contain both NAF and kinase domains, the characteristic features of CIPK proteins (Table 1). The 52 *CIPK* genes are distributed across 19 chromosomes (chromosome 1–20, except chromosome 12) (Table 1). These proteins range in size between 306 to 528 amino acids. The relative molecular weights of these CIPK kinase proteins varied from 35.14 to 59.71 kD. Most of these proteins (82.69%) have high isoelectric points (pI > 7.0). The detail information about other parameters was provided in Table 1.

### Phylogenetic and gene structural analysis of the soybean CIPK gene family

**Figure 1:**
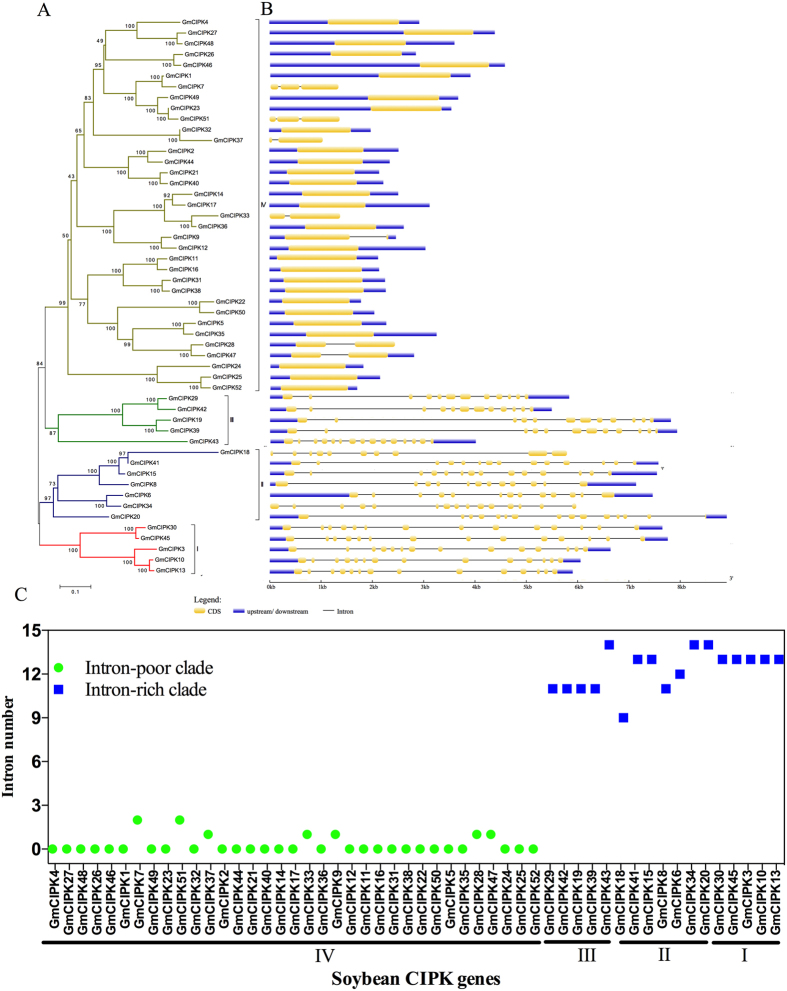
Phylogenetic relationship of soybean CIPK proteins and gene structure. The phylogenetic tree was generated using the MEGA6.06 software with the 52 full-length soybean CIPKs protein sequences (**A**). Neighbor-joining method was used with 1000 bootstrap replicates. These soybean *CIPK* genes were divided into four subgroups (I–IV) with different colored branches. Exon and intron analysis was performed using GSDS2.0 (**B**). The yellow boxes represent exons and the black lines represent introns. The blue boxes represent upstream/downstream-untranslated regions. The scale bars of introns, exons and untranslated regions are included at the bottom of the graph. (**C**) Classification of *CIPK* genes into intron-poor clade (green dots) and intron-rich clades (blue squares). Genes with intron number less than 3 were grouped into the intron-poor clade, and genes with intron number more than 8 were grouped into the intron-rich clade.

The evolutionary relationship among the 52 soybean CIPK members is shown in Fig. 1A. The phylogenetic analysis classified the 52 CIPK family members into four subgroups; I, II, III and IV (Fig. 1A). Subgroup IV is the largest one and contains 35 members. The other three subgroups contain 17 members in total (5 in subgroup I, 7 in subgroup II, and 5 subgroup III).

To further investigate the structural diversity of the *CIPK* genes in soybean, the exon/intron organization of the *GmCIPK* genes was analyzed. The *GmCIPK* gene members were clearly divided into an intron-rich clade (>8 introns per gene) and an intron-poor clade (<3 introns per gene)^23^,^26^. All the intron-poor clade members belong to subgroup IV and all intron-rich members relate to subgroup I, II and III (Fig. 1B). In subgroup IV, only *GmCIPK7* and *−51* contain two introns; *GmCIPK9*, −*28*, −*33*, *−37* and −*47* contain one intron; the other members in subgroup IV are intronless. Most members in subgroup III contain 11 introns except *GmCIPK43*, which contains 14 introns. Seven members in subgroup II varied in intron numbers from 9 (*GmCIPK18*) to 14 (*GmCIPK20* and −*34*). All genes in subgroup I contain 13 introns (Fig. 1C).

**Figure 2:**
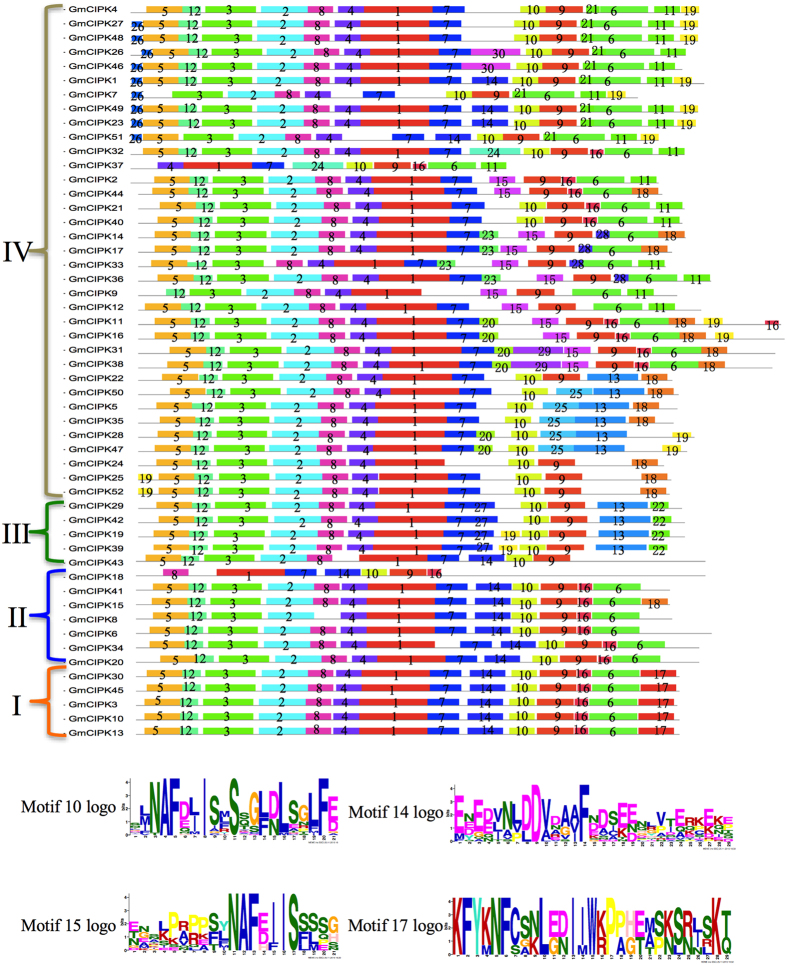
Conserved motifs in soybean CIPK proteins. The MEME program was used to investigate all relative motifs of soybean CIPK proteins. Each colored box represents a motif in soybean CIPK proteins. The relate name was on the left of each protein and the number of the motifs were showed in the boxes. Sequences logos of motif 10, 14, 15 and 17 were represented. Box length corresponded to motif length. Details of each motif were presented in Figure S1.

Conserved motifs were also analyzed for all the 52 soybean CIPK proteins using MEME software^33^. Totally thirty motifs were identified (Fig. 2) and the details of each motif were shown in Figure S1. All soybean CIPK proteins contained motif 10 or motif 15 annotated as the NAF domain. All proteins in subgroup I and II contain motif 14, but only 4 CIPK proteins in the subgroup IV, CIPK1, −23, −49 and −51, have motif 14. Only five proteins all in subgroup I, contain motif 17 (Fig. 2).

### Chromosomal location analysis and gene duplication

**Figure 3:**
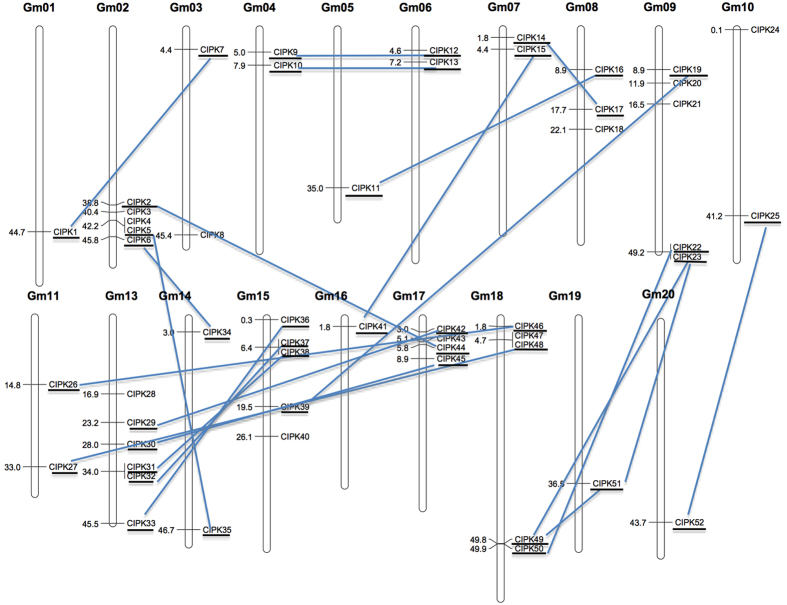
Chromosomal locations of soybean CIPK genes. The 52 soybean *CIPK* genes were mapped to 19 chromosomes. The duplicated *CIPK* gene pairs in the segmental duplicated blocks are underlined and connected by lines.

To determine chromosomal locations and duplication events, all the 52 *CIPK* genes were mapped to 19 out of the 20 soybean chromosomes, except chromosome 12 (Fig. 3). The 52 *CIPK* genes were not distributed evenly in these 19 chromosomes. Chromosomes 1, 5, 16, 19 and 20 contain one *CIPK* gene, while chromosome 13 contains most *CIPK* genes (6) among all soybean chromosomes.

Gene duplication events have driven the expansion of soybean *CIPK* genes, with 41 genes found in 22 duplicated blocks and only 11 *GmCIPK* genes located outside of the duplicated blocks (Fig. 3). Six pairs of genes, including *GmCIPK4/−5, 22/−23, 31/−32, 37/−38, 47/−48*, and *49/−50*, were separated by less than a 100-kb region on chromosome 2, 9, 13, 15 and 18, respectively, which were resulted from tandem duplications and were all intron-poor genes.

### Evolution analysis of CIPK in plants

**Figure 4:**
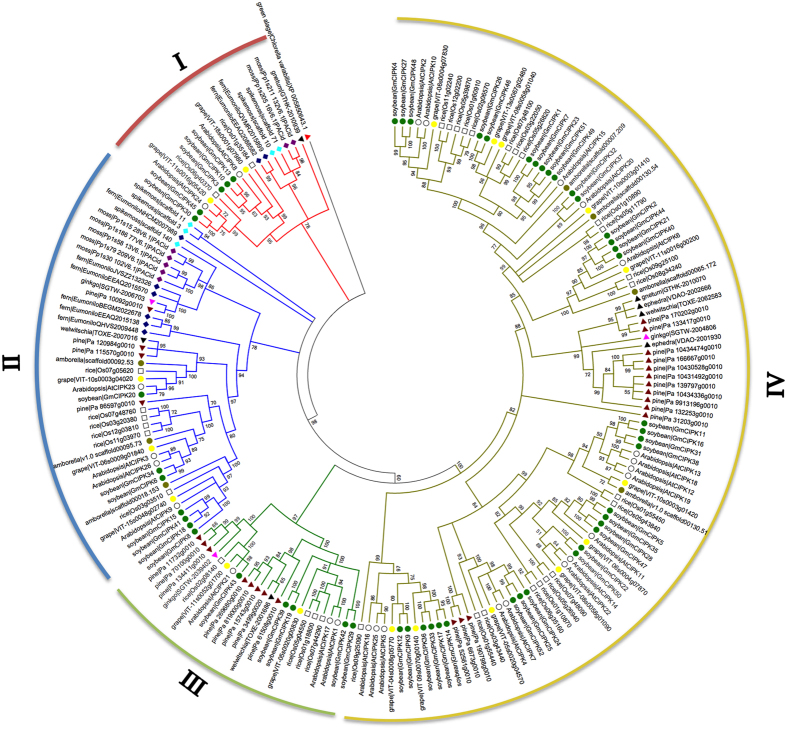
Evolution analysis of soybean CIPK proteins. The full-length of 193 CIPK protein sequences from soybean, grape, *Arabidopsis*, rice, amborella, ginkgo, pine, gnetum, ephedra, welwitschia, fern, spikemoss, moss and green algae were used to construct the phylogenetic tree using MEGA with the Neighbor-joining (NJ) method. Bootstrap values (on nodes) were calculated using 1000 replicates. Subfamilies are highlighted with different colors. The CIPK proteins in soybean were marked by green dots. A green algae CIPK protein was used as an outgroup and marked with a red triangle. Eudicot CIPK proteins were marked with dots. Monocotyledons were marked with squares.

To investigate the origin and evolution of CIPKs, we built a NJ phylogenetic tree using 193 full-length protein sequences containing a NAF (PF03822) domain from 14 representative plant species. Among the 193 proteins, only one CIPK protein was found in green algae, 7 in moss, 8 in fern, 5 in spikemoss, 26 in pine, 3 in ginkgo, 2 in gnetum, 3 in welwitschia, 2 in ephedra, 33 in rice, 7 in amborella, 18 in grapevine, 26 in *Arabidopsis*, and 52 in soybean. The CIPK proteins in rice, *Arabidopsis*, soybean and grapevine, all being angiosperm, were divided into four subgroups (Fig. 4). However, CIPK proteins in a green algae, moss, fern, and spikemoss were all grouped in subgroup I and II. All the 35 soybean intron-poor genes were clustered in subgroup IV (Fig. 1A). In addition, we found that some of gymnosperms plants (pine, ginkgo, gnetum, welwitschia and ephedra), amborella, rice, *Arabidopsis* and grapevine *CIPK* genes were assembled in subgroup IV. This result might represent plant intron-poor *CIPK* genes originated in seed plants.

### Global expression of soybean *CIPK* genes under drought

**Figure 5:**
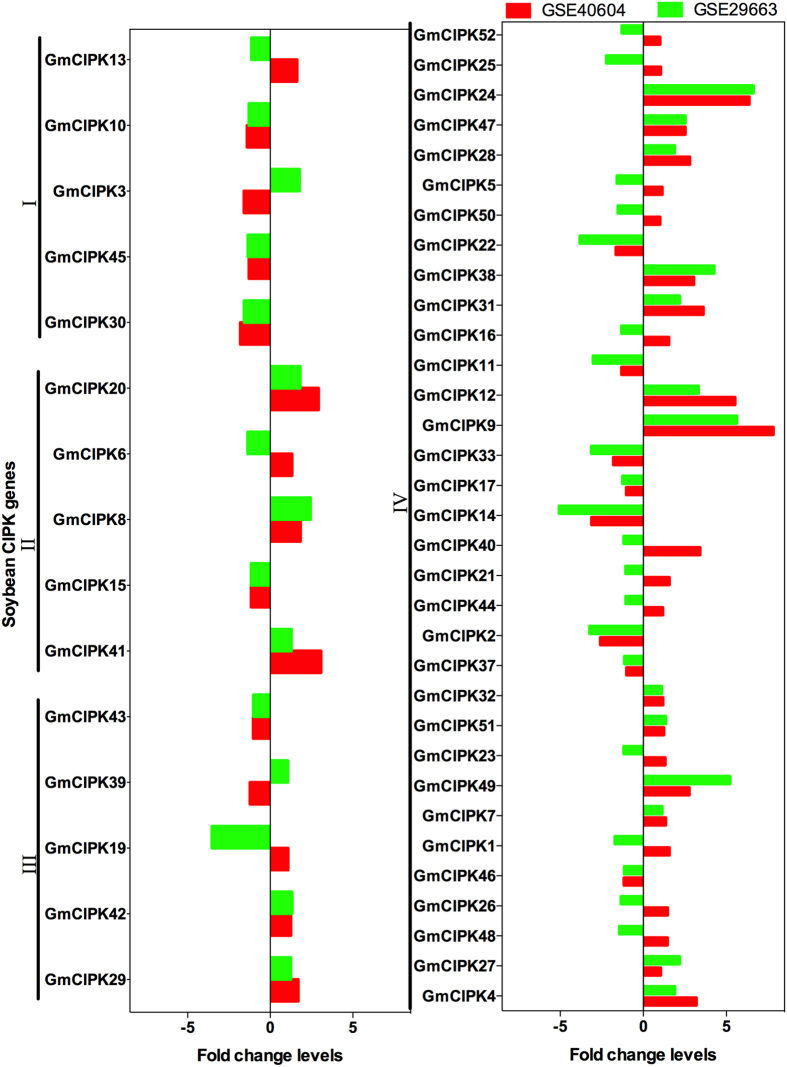
Expression levels of soybean *CIPK* genes under drought stress using publicly Affymetrix microarray datasets. Data are extracted from GSE29663 (green bars) and GSE40604 (red bars) datasets, which represent expression profiles in leaves under drought stress during early and late developmental stages, respectively.

To investigate gene expression change of the soybean CIPK family members under drought, we analyzed gene expression profiles of individual *CIPK* genes using publicly available Affymetrix microarray datasets. GSE29663 and GSE40604 datasets provide gene expression profiling of soybean leaves at early and late developmental stages under drought stress, respectively^9^. Gene expression data were available only for 48 *CIPK* genes, which have probes in this microarray platform (Fig. 5). Twenty genes were found to be up-regulated and 28 were down-regulated in the leaves during the vegetative growth stage in response to drought. During the reproductive stage, 33 were found to be up-regulated and 15 down-regulated, in the leaves in response to drought stress. Eighteen genes were up-regulated in both developmental stages under drought, including 3 in subgroup II (*GmCIPK8, 20* and *−41*), 2 in subgroup III (*GmCIPK29* and *−42*), and 13 in subgroup IV (*GmCIPK4, −7, −9, −12, −24, −27, −28, −31, −32, −38, −47, −49* and −*51*). In contrast, 13 genes showed constant down-regulation during both developmental stages. These genes included 3 in subgroup I (*GmCIPK10, 30* and *−45*), 1 in subgroup II (*GmCIPK15*), 1 in subgroup III (*GmCIPK43*), and 8 in subgroup IV (*GmCIPK2, −11, −14, −17, −22, −33, −37* and −*46*). The remaining 17 genes showed opposite expression patterns at both developmental stages (Fig. 5).

### qRT-PCR quantification of *CIPK* gene expression levels in leaves, stems and roots under drought stress

**Figure 6:**
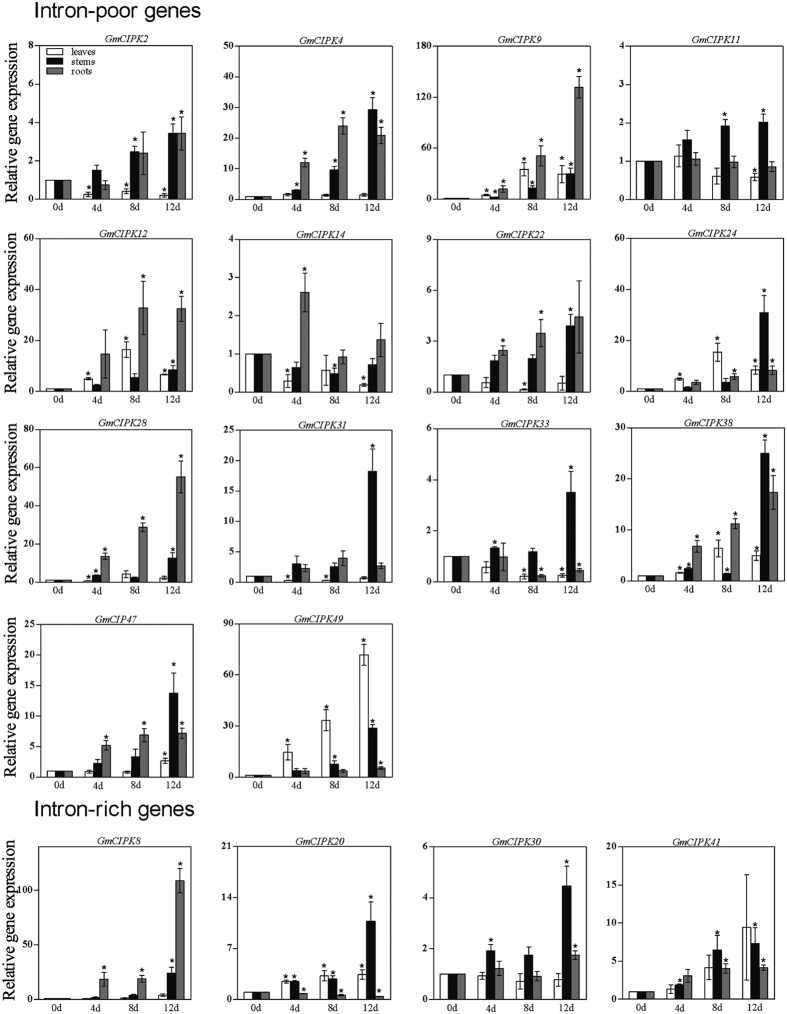
Quantification of gene expression levels of 18 selected soybean *CIPK* genes under drought stress using qRT-PCR. Fifteen-day-old plants were subjected to drought stress by withholding water for 0 (control), 4, 8 and 12 days. Leaf, stem and root samples were collected at these four time points for RNA extraction and qRT-PCR quantification of the expression levels of 14 intron-poor genes (top panels) and 4 intron-rich genes (bottom panels). *Ribosomal protein s20e* gene (*Glyma.03G142300*) was used as an internal control to normalize gene expression levels. The fold-change values represent changes of mRNA levels in drought stressed samples relative to non-stressed control samples. Data are average of three independent biological samples ± SE. Mean values significantly different from the control plants were determined by t tests (P < 0.05) and are indicated by an asterisk.

Based on the microarray data (Fig. 5), we selected 18 candidate genes (*GmCIPK2, −4, −8, −9, −11, −12, −14, −20, −22, −24, −28, −30, −31, −33, −38, −41, −47,* and *−49*) for further confirmation using qRT-PCR in leaves, stems and roots at 4-, 8-, and 12-days after imposing drought stress (Figure S2). The qRT-PCR results showed that all these 18 selected *CIPK* genes are drought-responsive and expressed differently in three different tissues under drought treatment (Fig. 6). In leaf samples, *GmCIPK2, −14, −31* and *−33* genes were down-regulated in the entire period tested under drought stress. However, *GmCIPK9, −12, −20, −24, −38,* and *−49* were up-regulated during all the three time points. Notably, *GmCIPK49* was induced gradually and showed the highest gene expression (>60-fold) after 12 days of drought application. Seven genes, including *GmCIPK9, −12, −20, −24, −38, −41,* and *−49*, were highly up-regulated at 8-day and 12-day time points. In the stem samples, *GmCIPK4, −8, −9, −24, −38,* and −*49* all showed higher gene expression levels (>20-fold) at 12-day than the untreated control. With the exception of *GmCIPK14,* which showed down-regulation, the remaining 17 genes exhibited up-regulation at the 3 time points tested. In root tissues, *GmCIPK9* showed the highest gene expression level at 12-day point, or 120-fold of that in the control. Most of these 18 genes were up-regulated at 8-day and 12-day drought treatment except *GmCIPK11, −14, −20 −30* and −*33*. While our qRT-PCR data are overall consistent with the microarray results (Fig. 5), a few genes displayed distinct expression patterns. This includes, for example, *GmCIPK31*, which was down-regulated in qRT-PCR assays of leave samples, but was up-regulated in the two microarray experiments.

## Discussion

Calcium plays a key role in plant signal transduction responding to environment stresses. Plant protein kinases such as calcium-dependent protein kinases (CDPKs) play central role in mediating plant response to stress signaling^39^,^40^. The calcium sensor calcineurin B-like proteins (CBLs) and their target kinase CBL-interacting protein kinases (CIPKs) system function together to regulate plant environmental stresses, such as drought^10^. *Arabidopsis* CBL1- and CBL9-CIPK23 complexes control abscisic acid (ABA)-regulated drought tolerance^41^.

CIPK family had been analyzed in some model plants and major crops^22^,^23^,^24^,^25^,^26^, but no detailed information about soybean CIPK gene family is available. In this study, we identified 52 *GmCIPK* genes in soybean (Table 1), which is twice as much as that in *Arabidopsis*, and more than that in most of the other plant species with sequenced Genomes. The large size of the CIPK gene family in soybean could be attributed to the whole-genome duplication events occurred approximately 59 and 13 million years ago (Mya)^1^.

The soybean CIPK gene family has significantly expanded in its evolutionary history. Similar to *Arabidopsis*^26^ and maize^23^, the soybean CIPK proteins were divided into four subgroups based on the phylogenetic classification (Fig. 1A). The soybean *CIPK* genes are clearly divided into intron-rich (subgroups I, II and III) and intron-poor (subgroup IV) clades (Fig. 1). This finding suggests that similar intron gain and loss events contributed to the structural evolution of the CIPK gene family before the eudicot–monocot divergence. All of the 52 GmCIPK proteins contain the signature NAF domain (Fig. 2)^13^. Our analysis of chromosomal locations and duplication events implicates gene duplication, especially segmental duplication and tandem duplication as the major evolutionary mechanisms responsible for soybean CIPK expansions (Fig. 3). Interestingly, all genes contributed by tandem duplication events were intron-poor genes. However, segmental duplication events occurred both in intron-poor genes and intron-rich genes, an observation similar to what was previously reported in *Arabidopsis*^26^. Tandem duplications have been found to be associated with gene families that regulate plant responses to stresses^42^, but little information is available with regard to the relationship between intron-poor gene clade and plant adaptation to environmental stresses.

Our phylogenetic analysis suggests that CIPKs originated in green algae, but expanded along the evolutionary trajectory to angiosperms. It is interesting that the intron-poor CIPK group was evolved much later, first appeared in the seed plants, very likely derived from loss of introns in the intron-rich members, as the CIPKs in the more ancient lineages, such as in green algae^14^, moss, fern and spikemoss were all in the intron-rich group (Fig. 4). This may suggests that when seed plants evolved, there was a great force for environmental stress adaptation. It has been previously reported that the intron-poor clade of the Hsp90 gene family in *Populus* displayed differential expression patterns upon exposure to various abiotic stresses, particularly drought stress^43^.

Plant *CIPK* genes could be induced by different stresses, such as drought^23^,^44^, salt^45^, and cold^18^. Various functional studies of plant *CIPK* genes provided clear evidence for their implication in stress responses. For example, overexpression of *SiCIPK24*(*SISOS2*) in tomato enhanced salt tolerance^46^. In Similarly, overexpression of *GhCIPK6* in *Arabidopsis* significantly increased the tolerance to drought, salt, and ABA^20^. Chaves-Sanjuan *et al.*^47^ described the CIPK protein structure and its regulatory mechanism during plant response to environmental stimuli. Consistent with a role of CIPK gene family in drought tolerance, a substantial number of soybean *CIPKs* changed mRNA abundance upon drought application as revealed by microarray analysis (Fig. 5). This was further verified in qRT-PCR assays (Fig. 6). The detailed gene expression analysis of soybean *CIPK* gene family in different tissues provided intriguing insight into their roles in responding to drought stress. Our qRT-PCR data showing differential expression patterns of 18 *CIPK* genes in three diverse tissues provide an indication of distinctive functional roles in of soybean CIPKs in different tissues in response to drought. In this context it may be important to mention that the unique and overlapping biological functions of *CIPK* gene family in different tissues are still unexplored^24^,^48^. Interestingly, we found the majority of drought-responsive *CIPK* genes in our qRT-PCR assays belong to the intron-poor gene clade in subgroup IV. This result suggests that expansion of intron-poor clade of *CIPK* genes may be an adaptive feature for drought stress^26^, but the mechanism underlying this adaptation remains elusive.

Among the up-regulated *GmCIPK* genes, we identified *CIPK9, −12, −24,* and −*49* as the most highly expressed genes under drought stress in the three tissues tested. These genes represent bona find targets for improving soybean tolerance to drought and deserve further analysis to reveal their functional roles in drought response and the underlying molecular mechanisms, which is currently underway.

## Additional Information

**How to cite this article**: Zhu, K. *et al.* Evolution of an intron-poor cluster of the CIPK gene family and expression in response to drought stress in soybean. *Sci. Rep.* **6**, 28225; doi: 10.1038/srep28225 (2016).

## Supplementary Material

Supplementary Information

## Figures and Tables

**Table 1:** List of 52 *CIPK* genes identified in soybean and their sequence characteristics.

Name	Gene ID (phytozome)	Chromosomal localization	Gene length (bp)	Amino acid length (aa)	PI	MW (kD)	Exons	CDS length (bp)
GmCIPK1	*Glyma.01g131500*	Gm01: 44789012–44792912	3901	467	8.69	52.85	1	1404
GmCIPK2	*Glyma.02g202900*	Gm02: 38802831–38805349	2519	430	9.10	48.36	1	1293
GmCIPK3	*Glyma.02g217300*	Gm02: 40416767–40423408	6642	444	6.11	50.45	14	1335
GmCIPK4	*Glyma.02g234800*	Gm02: 42228835–42231757	2923	463	8.85	52.87	1	1392
GmCIPK5	*Glyma.02g235100*	Gm02: 42255452–42257724	2273	442	7.12	49.25	1	1329
GmCIPK6	*Glyma.02g275900*	Gm02: 45882082–45889538	7457	472	8.06	53.77	13	1419
GmCIPK7	*Glyma.03g036900*	Gm03: 4497651–4498973	1323	413	8.63	46.46	3	1242
GmCIPK8	*Glyma.03g260200*	Gm03: 45407900–45415032	7133	440	8.45	49.93	12	1323
GmCIPK9	*Glyma.04g061500*	Gm04: 5015260- 5017719	2460	434	8.96	48.78	2	1305
GmCIPK10	*Glyma.04g090500*	Gm04: 7961747–7967803	6057	446	6.65	50.80	14	1341
GmCIPK11	*Glyma.05g158700*	Gm05: 35076361–35078476	2116	517	7.23	58.42	1	1554
GmCIPK12	*Glyma.06g062100*	Gm06: 4677276–4680311	3036	453	8.51	50.60	1	1362
GmCIPK13	*Glyma.06g092300*	Gm06: 7283361–7289264	5904	446	7.16	50.81	14	1341
GmCIPK14	*Glyma.07g023500*	Gm07: 1804676–1807191	2516	441	9.07	50.46	1	1326
GmCIPK15	*Glyma.07g051000*	Gm07: 4401020–4408559	7540	438	8.89	49.91	14	1317
GmCIPK16	*Glyma.08g116500*	Gm08: 8961418–8963555	2138	528	7.24	59.71	1	1587
GmCIPK17	*Glyma.08g218400*	Gm08: 17747795–17750917	3123	430	9.25	48.77	1	1293
GmCIPK18	*Glyma.08g252100*	Gm08: 22112310–22118088	5779	467	9.00	53.63	10	1404
GmCIPK19	*Glyma.09g079400*	Gm09: 8955776–8963586	7811	448	6.26	50.53	12	1347
GmCIPK20	*Glyma.09g089700*	Gm09: 11977305–11986209	8905	462	8.94	51.70	15	1389
GmCIPK21	*Glyma.09g098000*	Gm09: 16564068–16566202	2135	440	9.28	49.65	1	1323
GmCIPK22	*Glyma.09g276600*	Gm09: 49206095–49207881	1787	438	6.15	48.82	1	1317
GmCIPK23	*Glyma.09g277000*	Gm09: 49243593–49247135	3543	460	8.52	52.36	1	1383
GmCIPK24	*Glyma.10g001700*	Gm10: 165788–167599	1812	431	8.90	48.12	1	1296
GmCIPK25	*Glyma.10g179600*	Gm10: 41288232–41290368	2137	437	9.10	48.53	1	1314
GmCIPK26	*Glyma.11g161300*	Gm11: 14868303–14871145	2843	462	8.77	52.46	1	1389
GmCIPK27	*Glyma.11g235300*	Gm11: 33029240–33033627	4388	452	8.73	50.88	1	1359
GmCIPK28	*Glyma.13g069500*	Gm13: 16957654–16960090	2437	456	6.53	46.88	2	1371
GmCIPK29	*Glyma.13g119500*	Gm13: 23212628–23212628	5830	446	8.45	50.08	12	1341
GmCIPK30	*Glyma.13g166100*	Gm13: 28076313–28083963	7651	446	9.01	50.82	14	1341
GmCIPK31	*Glyma.13g228400*	Gm13: 34069476–34071724	2249	512	6.70	57.53	1	1539
GmCIPK32	*Glyma.13g228500*	Gm13: 34080809–34082788	1980	451	9.20	51.53	1	1356
GmCIPK33	*Glyma.13g370000*	Gm13: 45542607–45543980	1374	425	8.87	47.35	2	1278
GmCIPK34	*Glyma.14g040200*	Gm14: 3012174–3018140	5967	462	7.65	53.00	15	1389
GmCIPK35	*Glyma.14g203000*	Gm14: 46787303–46790554	3252	439	7.52	48.63	1	1320
GmCIPK36	*Glyma.15g003400*	Gm15: 309074–311683	2610	461	9.05	51.77	1	1386
GmCIPK37	*Glyma.15g084000*	Gm15: 6435171–6436213	1043	306	8.74	35.14	2	921
GmCIPK38	*Glyma.15g084100*	Gm15: 6445126–6447384	2259	510	6.75	57.27	1	1533
GmCIPK39	*Glyma.15g187400*	Gm15: 19503493–19511425	7933	437	7.58	49.17	12	1314
GmCIPK40	*Glyma.15g203700*	Gm15: 26165280–26167502	2223	438	9.31	49.51	1	1317
GmCIPK41	*Glyma.16g020200*	Gm16: 1828561–1835982	7422	438	9.05	49.86	14	1317
GmCIPK42	*Glyma.17g040700*	Gm17: 3002793–3008282	5490	448	8.75	50.39	12	1347
GmCIPK43	*Glyma.17g066300*	Gm17: 5105100–5109101	4002	467	8.82	52.82	15	1404
GmCIPK44	*Glyma.17g074800*	Gm17: 5871870–5874213	2344	422	9.17	47.60	1	1269
GmCIPK45	*Glyma.17g113700*	Gm17: 8997933–9005687	7755	446	9.06	50.83	14	1341
GmCIPK46	*Glyma.18g021600*	Gm18: 1584996–1589570	4575	449	8.99	50.31	1	1350
GmCIPK47	*Glyma.18g054600*	Gm18: 4749616–4752421	2806	450	6.18	50.12	2	1353
GmCIPK48	*Glyma.18g055000*	Gm18: 4773932–4777530	3599	462	8.80	52.40	1	1389
GmCIPK49	*Glyma.18g212200*	Gm18: 49872222–49875889	3668	462	8.85	52.65	1	1389
GmCIPK50	*Glyma.18g212700*	Gm18: 49942123–49944169	2047	443	6.41	49.71	1	1332
GmCIPK51	*Glyma.19g111300*	Gm19: 36555752–36557117	1366	426	7.99	48.84	3	1281
GmCIPK52	*Glyma.20g210800*	Gm20: 44730135–43731825	1691	436	9.22	48.62	1	1311

bp, base pair; aa, amino acids; PI, isoelectric point; MW, molecular weight; kD, kilo Dalton.
