# Supplementary material for: Evolution of an intron-poor cluster of the CIPK gene family and expression in response to drought stress in soybean
Source: Sci Rep. 2016 Jun 17;6:28225. doi: 10.1038/srep28225 (PMC4911590; doi:10.1038/srep28225)
Supplement: Supplementary Information [file srep28225-s1.pdf]

Evolution of an intron-poor cluster of the CIPK gene family and expression in response to drought stress in soybean

Kaikai Zhu, Fei Chen, Jinyi Liu, Xinlu Chen, Tarek Hewezi, Zong-Ming (Max) Cheng*

Department of Plant Sciences, University of Tennessee, Knoxville, Tennessee 37996, USA

*Corresponding author: zcheng@utk.edu

Supplementary information

Figure S1. Schematic diagram presentation of predicted motifs in the 52 soybean CIPK proteins.

Figure S2. Drought treatment affected the growth of soybean seedlings and soil water content.

Table S1. List of primer sequences used in qRT-PCR assays.

Figure S1

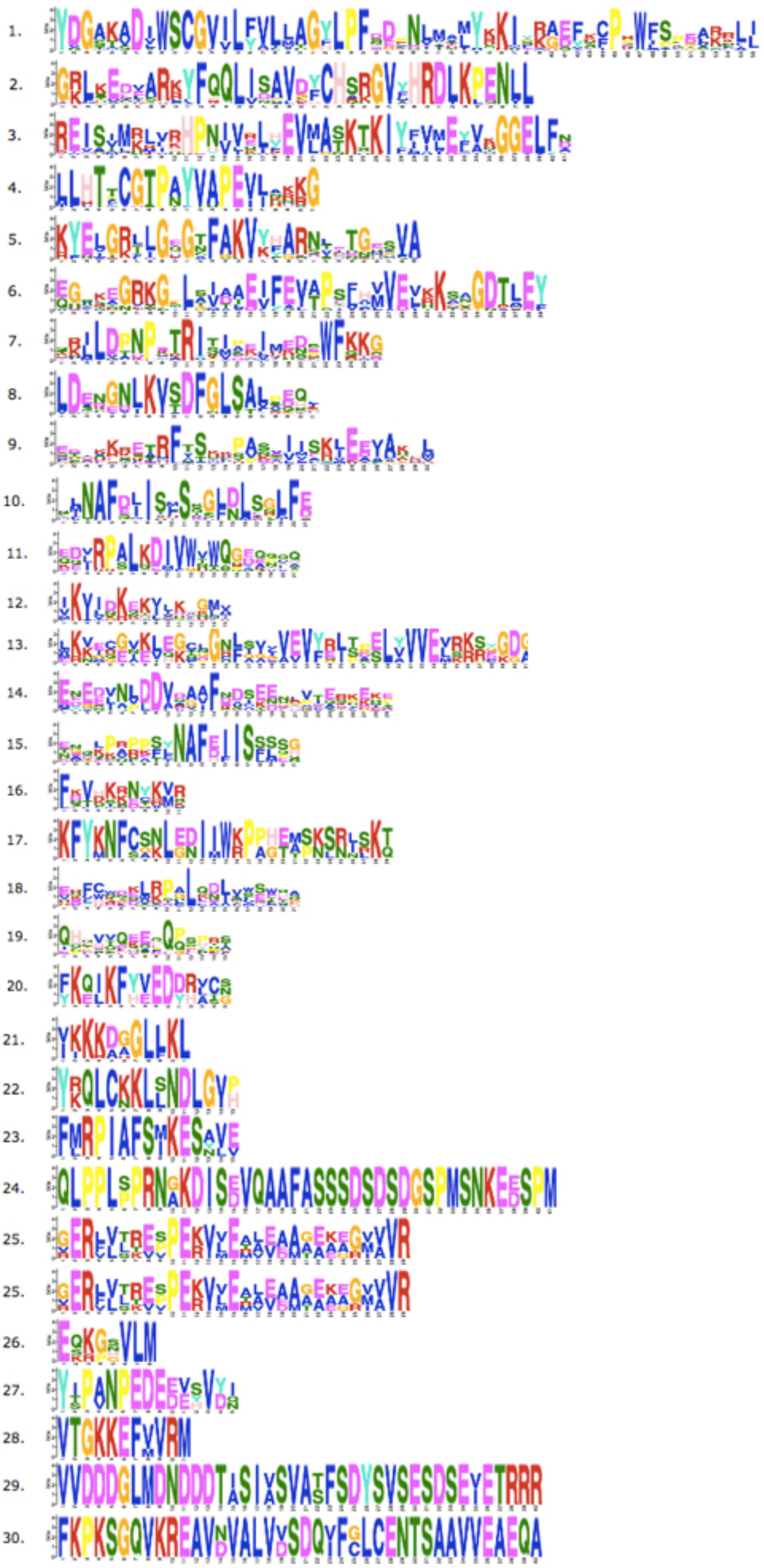

Figure S1. Schematic diagram presentation of predicted motifs in the 52 soybean

CIPK proteins. Motif prediction was performed using the MEME software suite.

Figure S2

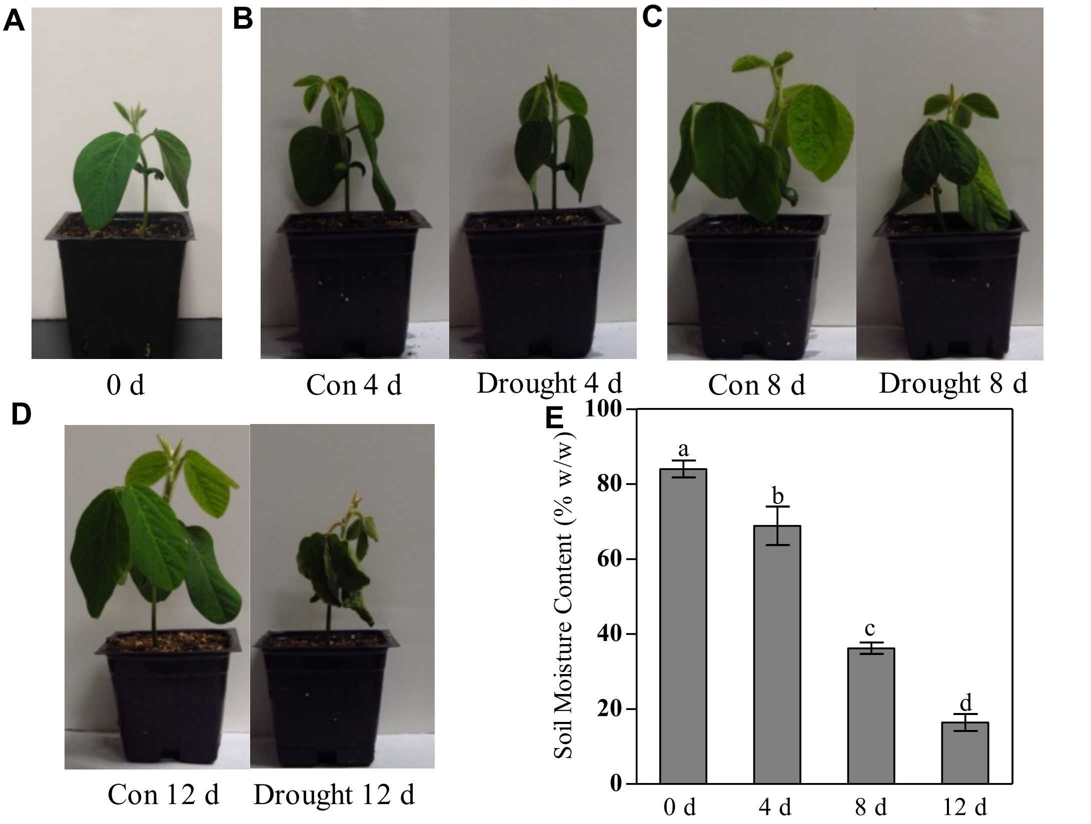

Figure S2. Drought treatment affected the growth of soybean seedlings and soil water content. Fifteen-day-old soybean seedlings were imposed to drought stress by withholding water for 0 (control, A), 4 (B), 8 (C), and 12 (D) days. (E) Soil moisture content at 0, 4, 8 and 12 day post drought application. Values are means ± SE of three independent experiments, each with three replicates. Bars with different letters signify statistically significant differences at $P < 0.05$ according to Duncan's multiple range tests.

Table S1. List of primer sequences used in qRT-PCR assays.

Gene	Sequence
GmCIPK2F	GGAGGTCAAGAAGGACAATG
GmCIPK2R	TGGGAGGAATTCCAGAAGA
GmCIPK4F	GGTGGAGGTGAAGAAATCAA
GmCIPK4R	CTTGCCAAACCCAAACAAC
GmCIPK8F	TCTTACTACTGCTCGACTTAGA
GmCIPK8R	CCCTAGAGGTTGCTTAATTCTT
GmCIPK9F	AATACGCCAAGTTCTGTGAG
GmCIPK9R	CCCTGACTTGCTCTTTCAC
GmCIPK11F	GGTGAATCAAAGTTCGAATGTC
GmCIPK11R	GTCATTCTCCTGCTACCTTAAT
GmCIPK12F	CTCGAATATGACAGATGGGAAC
GmCIPK12R	GGATGCTTGGAGCTGTATTT
GmCIPK14F	TTGTGACGGTGTTTGATTTG
GmCIPK14R	CCAGCCAACTGAAACTCTAA
GmCIPK20F	TCTCCGTACGAAGTACAGTC
GmCIPK20R	ATCCTCCAATACCCACTCTT
GmCIPK22F	GATTTGGAAGAGCCGGTATC
GmCIPK22R	ACCCTTGAGTAAACACCAATC
GmCIPK24F	TGTGGTTGAGGTGTTTGAG
GmCIPK24R	CATGCCATGAGAGAACAAGA
GmCIPK28F	GAGAGGTTGAAGCCATTGT
GmCIPK28R	CTCAGGTTCTTGTTCCTGTT
GmCIPK30F	CTAGCTATCATAATGCAACATACAC
GmCIPK30R	TCCTTTCCTTCTCCCAAGA
GmCIPK31F	ATTGCAGGGTGAGTTTGG
GmCIPK31R	CTCCACCACAACCAAAGAA
GmCIPK33F	CACCAGTAACGCAATGGA
GmCIPK33R	AAGCTTGGAGGAGGAAATAAG

GmCIPK38F	CCTACTGATGATTGAAGACTGAG
GmCIPK38R	GAGAGAGAGAGAGAGAGAAA
GmCIPK41F	CGCCCTGCAAATGAAATC
GmCIPK41R	ACCCTTCCTTCCACTCTTA
GmCIPK47F	TTTGAATGGCTTGGGTTAAATC
GmCIPK47R	CCTTCCAACTTCCATCCATC
GmCIPK49F	CCGGCCTCAATCATAATCTC
GmCIPK49R	TTCCTGCCTTCTTTGGAAC
RSP s20eF	GATGCCCACTAAGGTTCTTC
RSP s20eR	GTGCACACGAAGTTCAAATC
